# Tendency of Semaglutide to Induce Gastroparesis: A Case Report

**DOI:** 10.7759/cureus.52564

**Published:** 2024-01-19

**Authors:** Ahtshamullah Chaudhry, Buluku Gabriel, Jawad Noor, Saima Jawad, Suryanarayana R Challa

**Affiliations:** 1 Internal Medicine, St. Dominic Hospital, Jackson, USA; 2 Internal Medicine, Nishtar Medical University, Multan, USA; 3 Gastroenterology, Howard University Hospital, Washington, USA

**Keywords:** nausea and vomiting, upper endoscopy, weight loss and obesity, semaglutide, gastroparesis

## Abstract

Semaglutide, an agonist of the glucagon-like peptide-1 receptor, is frequently used in the treatment of diabetes mellitus type 2, although, lately, weight loss has additionally become a reason for its use. However, if a patient is already experiencing bloating, nausea, abdominal pain, and discomfort in the abdomen, it is not recommended to use it due to concern about aggravating these symptoms. Although it is often well tolerated, there are occasions when it can have several gastrointestinal side effects. Therefore, we report a case of a patient who started taking semaglutide and later developed gastroparoresis.

## Introduction

Gastroparesis is a condition of mechanically unhindered, delayed stomach emptying. It causes upper abdominal discomfort, bloating, nausea, early satiety, and postprandial fullness [1]. One of the well-known long-term adverse effects of diabetes is gastroparesis, which is responsible for almost one-third of all cases [2]. Semaglutide is primarily used for the treatment of diabetes mellitus type 2 [3]. There is already a concern about semaglutide being associated with gastroparesis [4].

A diagnosis of gastroparesis can be made by ruling out mechanical obstruction and presence of delayed stomach emptying. The goal of treatment for gastroparesis is to provide symptomatic relief by using prokinetic agents while ensuring adequate nutrition through small frequent meals until resolution [5]. Therefore, this case portrays drug-induced gastroparesis as one of the differentials after ruling out common causes of gastroparesis.

## Case presentation

A 53-year-old woman with a medical history of depression, obesity stage 2, and obstructive sleep apnea presented to the hospital due to nausea and abdominal pain for the last three weeks. She also reported a weekly intramuscular injection of 0.5 mg of semaglutide for the preceding four months for weight loss, with an estimated 40-pound loss during this period. She did not escalate the dose of semaglutide for weight loss as recommended, as she was satisfied with the response to the initial dose. Her other home medications included alprazolam, methocarbamol, desvenlafaxine, and bupropion.

On admission, her blood workup showed serum sodium of 136 mmol/L, bicarbonate of 25 mmol/L, potassium of 4.3 mmol/L, aspartate aminotransferase (AST) of 30 U/L, alanine aminotransferase (ALT) of 29 U/L, lipase of 23 U/L, C-reactive protein of 0.9 mg/L, white blood cell count of 7.5 x 10^3^/µL, thyroxine stimulating hormone of 0.876 uIU/mL (normal value: 0.4 to 4.0 mIU/L), and hemoglobin A1C of 4.8 (normal: below 6.5). The abdominal examination was significant for mild abdominal discomfort without rigidity, guarding, and rebound tenderness with normal bowel sounds.

There was a concern for abdominal obstruction; hence, a computed tomography scan of the abdomen and pelvis was ordered, which showed no external obstruction and no other acute pathology. The patient also had a negative antinuclear antibody, and her immunoglobulin M level was 103 mg/dL and immunoglobin G level was 409 mg/dL. Diabetes mellitus, intraluminal and extraluminal obstruction causes, and autoimmune processes were ruled out with the above negative results.

The patient was started on IV ondansetron 4 mg every 6 hours as needed for nausea, and she was able to tolerate a semisolid diet. She underwent an endoscopy the following day, which revealed a normal esophagus and semisolid food in the fundus; hence, the procedure was aborted due to retained food material. The patient was placed on clear liquid diet and was advised to stop drinking after midnight and proceeded to undergo a repeat esophagogastroduodenoscopy (EGD) the following day, which still showed a small volume of retained semisolid food material and liquid in the cardia and fundus of the stomach.

This finding of retained food material in the stomach on upper endoscopy more than 24 hours apart was considered highly consistent with the diagnosis of gastroparesis. The gastroenterology service attributed this to the use of semaglutide and recommended that the patient stops its use. A gastric emptying study, which is the gold standard for diagnosis of gastroparesis, was not performed as the gastroenterologist had a high degree of certainty of the diagnosis given symptoms and findings on EGD. The patient reported significant improvement in symptoms at one-month follow-up after stopping semaglutide, with complete resolution of nausea.

## Discussion

Gastroparesis is a chronic disorder that is characterized by delayed stomach emptying without mechanical blockage. An estimated 5 million people in the United States of America suffer from some type of gastroparesis [2]. In this case, the patient's use of semaglutide and her symptoms of gastroparesis seem to be strongly related. By stimulating glucagon-like peptide-1 (GLP-1) receptor agonists, it increases incretin action. Furthermore, better blood glucose levels are achieved by increased glucose-dependent insulin secretion, decreased hepatic gluconeogenesis, delayed gastric emptying, and inhibited glucagon release [6]. Additionally, semaglutide encourages weight loss by delaying gastric emptying and decreasing energy intake [6]. Semaglutide mainly binds to plasma proteins, has a half-life of approximately one week, and remains in circulation for about five weeks after the last dose. Its effects increase in a dose-proportional manner, and a steady state is achieved after four to five weeks of once-weekly administration.

The exact mechanism by which semaglutide may cause gastroparesis is unknown. Nonetheless, it is well recognized that GLP-1 receptors regulate stomach emptying and motility [3,6]. Therefore, the stimulation of these receptors may cause gastroparesis by delaying the stomach's emptying. Proton pump inhibitors, opiates, GLP-1 receptor agonists, and anti-Parkinson's drugs are among the other drugs that can delay gastric emptying [4]. In one trial, a healthy volunteer taking GLP-1 antagonists compared to a placebo saw a substantial delay in stomach emptying [7].

Proof of delayed gastric emptying in gastric emptying studies is required for the diagnosis, in addition to the absence of mechanical blockage. Consequently, upper and occasionally lower endoscopies are frequently included in the workup [8]. The decision to forgo a gastric emptying study for our patient was made due to financial concerns and clinical evidence observed during repeated endoscopy, which included the presence of food material in the stomach 24 hours apart and the absence of mechanical blockage [9]. A gastric emptying study is considered positive for gastroparesis if there is more than 60% of retaining food after 2 hours or 10% after 4 hours [10]. Kalas et al. reported a study that was conducted on healthy individuals and showed delayed gastric emptying in 50% of healthy participants [11]. Moreover, the precise prevalence of gastroparesis caused by semaglutide is still unknown.

The mainstay of therapy for gastroparesis includes replacement of electrolytes, hydration, and dietary changes (eating small, frequent meals with less fat and containing soluble fibers), as well as identifying and eliminating the triggering etiology [12]. Most of the time, stopping semaglutide leads to improvements in nausea, stomach pain, and vomiting [2]. This finding has led to the recommendation by the American Society of Anesthesiologists to hold GLP-1 agonists prior to elective surgery (one week in advance) to prevent nausea and vomiting that may occur during the induction of anesthesia and intubation [1].

## Conclusions

Although the gastrointestinal adverse drug reactions of semaglutide are well known, gastroparesis is unusual. The fact that patients' symptoms significantly improved once the use of Semaglutide was discontinued highlights the need to recognize medication-induced gastroparesis as a possible diagnosis, especially in individuals with risk factors.
